# Conversion of Waste Oil from Oil Refinery into Emulsion Liquid Membrane for Removal of Phenol: Stability Evaluation, Modeling and Optimization

**DOI:** 10.3390/membranes12121202

**Published:** 2022-11-29

**Authors:** Dan Wang, Qingji Wang, Xiaofei Zhang, Taoran Liu, Hua Zhang

**Affiliations:** 1 State Key Laboratory of Petroleum Pollution Control, Beijing 102206, China; 2 CNPC Research Institute of Safety and Environmental Technology, Beijing 102206, China; 3 Petrochemical Research Institute, PetroChina, Beijing 102206, China

**Keywords:** waste oil reuse, waste oil emulsion liquid membrane, refinery wastewater, model establishment

## Abstract

The waste oil emulsion liquid membrane produced by waste oil from oil refineries (WELM) is used to separate the phenol in purified water from the sour water stripper in oil refinery facilities, and the stability of WELM was studied. It is verified that waste refinery oil can be produced into emulsion liquid membrane with good stability and high removal rate for the first time. The WELM stability models were established by response surface methodology (RSM) and artificial neural network (ANN), respectively. The principle and mechanism of various parameters, as well as the interaction effects on the stability of WELM, are proposed. The effects of parameters, including the ratio of Span-80, liquid paraffin, the ratio of internal and oil, and the rotational speed of the homogenizer, were investigated. Under the optimal operating parameters, the WELM had a demulsification percentage of just 0.481%, and the prediction results of RSM and ANN were 0.536% and 0.545%, respectively. Both models demonstrate good predictability. The WELM stability model has a high application value in the treatment of phenol-containing wastewater in the oil refining industry, and provides a green method of resource recovery.

## 1. Introduction

Liquid membrane technology is a technique that integrates extraction and separation, as well as extraction and back-extraction. The liquid membrane has selective permeability as the separation process medium, and it separates the liquid phase into two parts, with the concentration difference of substances between the two parts acting as an impetus to separate the substances. Bulk liquid membrane (BLM), supported liquid membrane (SLM), and emulsion liquid membrane (ELM) are the three primary types of liquid membrane. SLM and ELM are often employed in the field of wastewater treatment.

**Figure 1:**
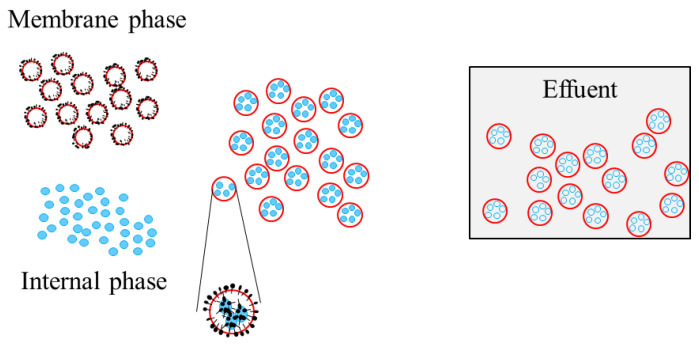
Schematic diagram of W/O-type emulsion film membrane structure.

Since ELM was invented in 1968, it has caught the interest of researchers from a variety of fields. Many studies on ELM separation in the field of environmental protection have been conducted in recent years, due to the rising severity of environmental contamination. Because the ELM technology has low energy consumption, minimal pollution, low chemical dosage, and cheap maintenance cost, it has a great industrial application potential. The primary types of ELM are oil-in-water-in-oil (O/W/O) and water-in-oil-in water (W/O/W). W/O/W is commonly used to extract organic contaminants from water. Figure 1 illustrates the structure of ELM. ELM consists of three parts, including membrane phase, inner phase, and the outer phase. A variety of chemicals, including film solvents, surfactants, and film auxiliaries, are mixed to produce the film phase. The part wrapped inside the membrane phase is the inner phase that extracts pollutants [1]. The wastewater to be separated is the outer phase. The W/O/W ELM exists as spherical droplets in the outer phase. The different affinities of the surfactant to the oil and water phases form the emulsion membrane [2]. The substances to be separated in the outer phase are transferred into the inner phase via the emulsion membrane and react with the chemicals in the inner phase, producing salts that cannot pass through the membrane back to the outer phase, resulting in substance separation and concentration. Demulsification recovers the separated compounds in the ELM, and the membrane phase can be recycled [3]. One of the most important factors effecting the separation is the stability of ELM. In the ELM production process, stability is related to the mechanics properties, raw material properties, and operational characteristics. As a result, research on ELM stability is the foundation and crucial to the development of ELM technology and its industrial application [4].

Raja Sulaiman et al. produced an ELM to extract ionized silver nanoparticles, and they investigated the effects of various parameters on the stability and separation, such as pH of outer phase, carrier concentration, stripping agent concentration, emulsification time, homogenizer rotational speed, separation speed, and surfactant concentration [5]. The results demonstrated that 99.89% of silver ions could be extracted under the optimal conditions of a pH of 2, the Cyanex 302 concentration of 0.0005, the acidic thiourea concentration of 1.0 M, emulsification time of 5 min, homogenizer rotational speed of 12,000 rpm, and separation stirring speed of 250 rpm. The addition of ionic liquid [BMIM]^+^[NTf2] to the membrane phase can increase emulsion stability and minimize solution swelling by about 10%. Khalil Abbassian et al. studied ELM stability and concluded that it is the most crucial condition for a successful ELM production [6]. They introduced EPDM (poly ethylene-propylene diene monomer) as a non-Newtonian modifier to the phenol-containing membrane phase separation solution and discovered that adding EPDM can effectively improve the stability of liquid membrane and treatment effect.

Sadana Sujatha produced environmentally friendly green emulsion liquid membrane (GELM) from waste cooking oil (WCO) as diluent for Pb extraction [7]. The optimal preparation conditions were obtained by Box-Behnken Design (BBD). The researchers found that the GELM could be recycled eight times under certain conditions, with the membrane stability remaining almost constant for the first six times and a maximum Pb extraction of 97.39%. Norasikin Othma et al. investigated the use of palm oil as a diluent in the production of emulsion membrane to treat simulated wastewater with the phenol concentration of 300 × 10^−6^ mol/L [8]. The effect of emulsification rotational speed, emulsification time, surfactant concentration, and stirring speed on the stability of ELM were investigated. It was found that when the emulsification rotational speed is 1300 rpm, emulsification time is 5 min, stirring speed is 500 r/min, and surfactant concentration is 3%, the probability of swelling and rupture of the liquid membrane is low, and the ELM stability is comparatively best. Parastoo Tahmasebizadeh et al. studied that ELM was used to extract zinc from bioleaching of low-grade lead/zinc sulfide ores [9]. It was found that excessive shear forces, additional concentration of emulsion components, and longer contact times were observed to induce emulsion cracking and pellets breaking. Varun Arun et al. investigated the effect of various surfactants (including Span-80, BKC, and CTAB) on the stability of ELM and phenol removal rate [10]. Some studies found that with the increase in surfactant concentration, the viscosity of the emulsion, which leads to an increase in the resistance of pollutants to diffusion through the emulsion, and further experiments demonstrated that 3 wt.% Span-80 was adequate to produce stable emulsion [11,12]. Huda M. Salman et al. investigated the effect of magneticα-Fe_2_O_3_ particles on the stability of kerosene emulsion liquid and Pb in the solution was separated. The research found that when the homogenizer rotational speed was 12,700 rpm, the dosing concentration of Span-80 was 2% *v/v*, the carrier concentration was 4% *v/v*, the concentration of Fe_2_O_3_ was 0.3% *w/v*, the pH of the solution was 3, and the separation speed was 250 rpm, while the recycling rate of Pb was 97.2% after 10 min of emulsification and 8 min of contact separation, whereas the demulsification rate was just 0.3% [13].

The novelty of this study lies in the use of waste oil-based emulsion liquid membrane to removal of phenol in oil refinery wastewater and the RSM and ANN models of emulsion liquid membrane stability were established. Through the established models, the stability can be predicted under a certain condition, and the preparation conditions can be determined according to production demand. In this study, a waste oil-based ELM (WELM) is formulated by using oily sludge hydrothermal treatment flash light oil as an alternative to membrane solvent to remove phenol from the purified water of the sour water stripper in oil refinery facilities. There are several advantages of WELM, including its lack of secondary pollutants, low cost, waste recycling, and environmental conservation [14,15]. The utilization of waste oil as a substitute for membrane solvent demonstrates a path for transforming low-value waste items into beneficial raw materials for ELM synthesis. The experiments were designed and carried out by using the Box-Behnken Design (BBD) method, the data were processed by using Response Surface Method (RSM) and Artificial Neural Networks (ANNs), and the prediction models of WELM stability were established, which provide theoretical foundation for the utilization of WELM, as well as the subsequent demulsification and recycling of the membrane phase.

## 2. Materials and Methods

### 2.1. Experimental Procedure

The experimental procedure is separated into two parts: production of WELM and contact separation. In the section of WELM preparation, a homogenizer that can create considerable shear force and produce stable emulsion was employed. A magnetic stirrer was applied as a stirring device in the contact separation section. The object to be treated was purified water of the sour water stripper in an oil refinery facility, with volatile phenol concentration of around 370 mg/L, and the concentration range of phenol is 170–180 mg/L, and COD of about 1200 mg/L.

**Figure 2:**
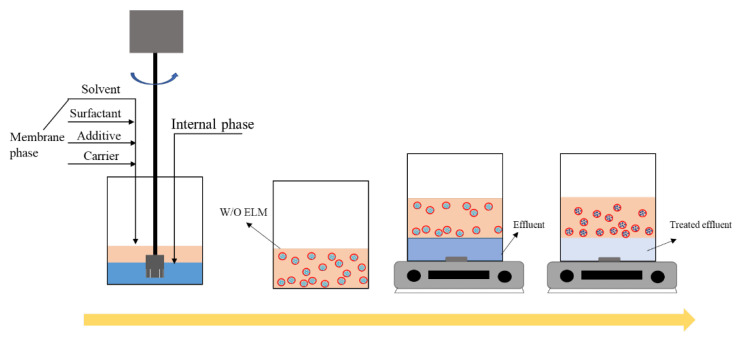
Flow chart of the separation experiment of emulsion liquid membrane production.

The experimental process is shown in Figure 2. A certain quantity of NaOH (AR, Shanghai HUSHI laboratory equipment Co., Ltd., Shanghai, China) is weighed and dissolved in ultrapure water as the internal phase. The surfactant, membrane solvent, carrier, and film additive are weighed as the raw material of the membrane phase. In this study, waste oil is a kind of flash light oil, which was taken from the oily sludge hydrothermal treatment in an oil refinery. The properties of the waste oil in this study are shown in Table 1. Span-80 (AR, Shanghai test) is selected as the surfactant, waste oil as the film solvent, liquid paraffin (AR, Shanghai test) as the film additive, tributyl phosphate (TBP) (AR, Shanghai test) as the carrier. Surfactant, membrane solvent, carrier, and film additive are emulsified in a homogenizer (FJ200-SH, Shanghai Huxi Industrial Co., Ltd., Shanghai, China), and the NaOH solution is slowly introduced in the early stages of emulsification. Finally, the WELM is obtained after a period of homogenization.

The WELM and wastewater to be treated are mixed, and contact stirring is conducted using a magnetic stirrer (MS-H280-Pro, DLAB Scientific, Beijing, China). After stirring, the liquid is placed into a separatory funnel and left to stratify. After certain amount of time, the upper layer liquid is the utilized emulsion, while the bottom layer is the treated waste liquid. The lower layer liquid was sampled by ICP (Full Spectrum Direct Reading Inductively Coupled Plasma Emission Spectrometer, Optima 7000 DV, Perkin Elmer instruments, Waltham, MA, USA) to analyze the concentration of Na^+^ ions. The WELM will swell and coalescence owing to instability during the separation process, resulting in the Na^+^ in the internal phase entering to the outer phase, and the concentration of Na^+^ detected in the outer phase indicates the WELM demulsification. The expression is shown in the Equation (1). The demulsification rate of ELM is represented with S. The concentration of Na^+^ in the external phase before and after separation are C(Na^+^)_out-0_ and C(Na^+^)_out-1_, respectively, and *V* as the volume of the bottom liquid, and N(Na^+^)all as the total quantity of Na^+^.

(1) $$S(\%)=\frac{{(C(\mathrm{Na}}^{+}{)}_{\mathrm{out}-1}-{C(\mathrm{Na}}^{+}{)}_{\mathrm{out}-0})\;\times \;V}{{N(\mathrm{Na}}^{+}{)}_{\mathrm{all}}}\;\times \;100\%$$

### 2.2. RSM Model Development and Analysis

Design-Expert is used for experiment design, model establishment, and result analysis. The experimental design was developed with the Box-Behken (BBD) approach, and a 4-factor 3-level experiment was established. According to the literature, four factors have a significant effect on the stability of ELM, including the dosage of Span-80, the dosage of liquid paraffin, the proportion of internal and membrane, and the rotational speed of homogenizer. Table 2 displays the specific factor level values. The BBD design method was used to design 29 groups of four parameters and three levels of experiments. Table 3 shows the experimental results. The response surface analysis approach was used to examine 29 sets of data, and among several fitting equations, the quadratic equation (such as Equation (2)) with the least error and highest fitting degree was adopted as the prediction equation.

(2) $$S=\alpha_{0}+\overset{4}{\underset{i=1}{\sum }}\alpha_{i}Z_{i}+\overset{4}{\underset{i=1}{\sum }}(\overset{4}{\underset{j=i}{\sum }}\alpha_{ij}Z_{i}Z_{j})+\overset{4}{\underset{i=1}{\sum }}\alpha_{ii}Z_{i}{}^{2}$$

In the fitting equation, *S* is the predicted response value, *Z* is a variable and *α*_0_, *α_i_* (*α_j_*) and *α_ii_* are the offset, linear coefficient, two-factor interaction coefficient, and quadratic coefficient, respectively. The RSM model was established and evaluated by analysis of variance (ANOVA), and the response surface model diagram was utilized to investigate variable interaction.

### 2.3. ANN Model Design and Analysis

In the last two decades, artificial neural networks (ANN) have garnered considerable attention. It excels at modeling, optimization, simulation, and prediction of system performance. ANN is characterized by its great processing speed and accuracy. There are several ANN models, the most common of which are the BP neural network, RBF neural network, SOM neural network, Hopfield neural network, FNN feedforward neural network, and others. BP and RBF neural networks are the most typically employed predictive models.

**Figure 3:**
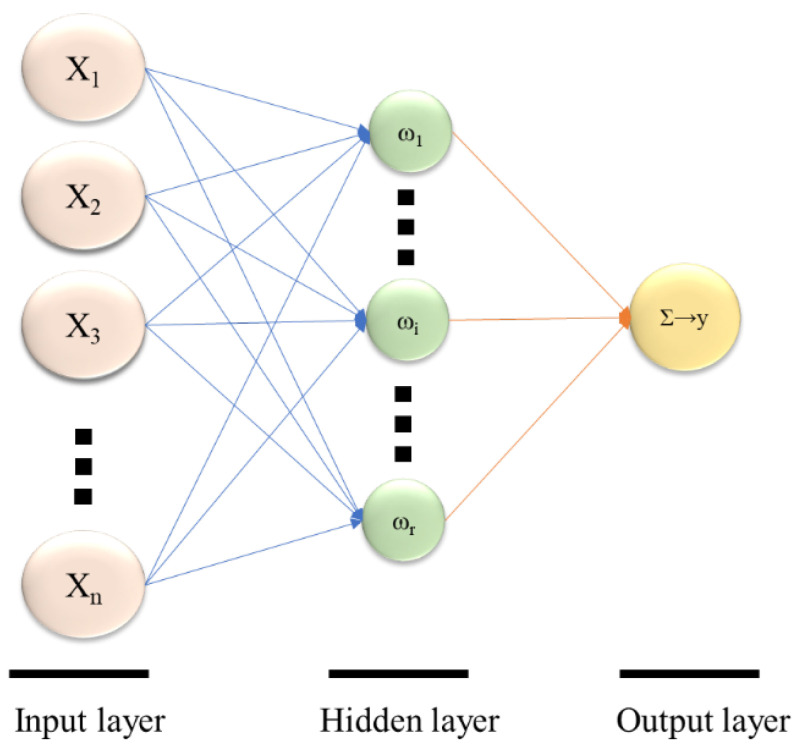
Structure of ANN model building.

The Neural Net Fitting toolbox in Matlab software (R2019b, MathWorks, Natick, MA, USA) was used to construct the ANN (Artificial Neural Net) model in this study. Using the same experimental data as the BBD design, the experimental data were modeled. As shown in Table 3, 75% of them are utilized as model building data, 15% as test data, and 15% as test data. Figure 3 depicts the model construction procedure. The model is made up of three layers: an input layer, an output layer, and a hidden layer. The hidden layer has numerous nodes, with the number defined by Equations (3) and (4) [16].

(3) $$\frac{{2(n}_{i}{+n}_{o})}{3}{\;<\;N\;<\;n}_{i}\left(n_{i}{+n}_{o}\right)-1$$

(4) $$0{.5n}_{i}-{2\;<\;N\;<\;2n}_{i}+2$$

The numbers *n*_i_ and *n*_o_ in the equation represent the number of input and output layers, respectively, and *N* represents the number of hidden layer nodes. The network with the fewest hidden layer nodes and the greatest performance in the test set was chosen within a certain range. Because modifying parameters implies updating the neural network, which complicates the process of establishing the best number of hidden layer nodes, it is essential to maintain other specified parameters constant when testing the number of hidden layer nodes in the network.

The weight of the connection between the input layer and the hidden layer is *σ*i. The key to building a neural network model is obtaining weight values. The neural network’s input and hidden layers, as well as its output and hidden layers, may learn any mapping function. The output of the hidden layer and the output layer of the FNN neural network and the BP neural network are expressed as follows.

(5) $$z_{m}=f\left({net}_{m}\right)=f(\underset{l}{\sum }\sigma_{lm}x_{l}-\theta_{m})$$

(6) $$y_{n}=\Phi \left({\mathrm{net}}_{n}\right)=\Phi (\underset{n}{\sum }\varphi_{\mathrm{mn}}y_{m}-\theta_{n})\;$$

*σ* and φ are the weights of input and output layer, respectively. *x*_l_, *y*_m_, and *z*_n_ are the vector of input, output, and output of hidden layer, respectively. θ_m_ and θ_n_ are neuron threshold and *f*(net_m_) and Φ(net_n_) are activation function.

In this study, coefficient of determination (R^2^), root mean square error (RMSE) and mean absolute percentage error (MAE) were selected to evaluate the merits of the neural network model.

(7) $$R^{2}=1-\frac{SSE}{SST}=1-\frac{\sum {{(u}_{i}-\hat{u_{i}})}^{2}}{\sum {{(u}_{i}-\;\bar{u})}^{2}}$$

(8) $$\mathrm{RMSE}=\sqrt{\frac{\sum {\left(u_{i}-\hat{u_{i}}\right)}^{2}}{n}}$$

(9) $$\mathrm{MAE}=\frac{1}{n}\sum |u_{i}-\hat{u_{i}}|\times 100\%$$

*u*_i_ and $\hat{u_{i}}$ are the model predicted and experimental values, respectively.

## 3. Results and Discussion

### 3.1. RSM Modeling and Forecasting Performance Evaluating

The response value of the demulsification rate can be represented as a quadratic equation using RSM to assess the experimental results, as shown in Equation (10).

The dosage of Span-80, liquid paraffin, the ratio of internal phase to oil phase, and the rotational speed of homogenizer are independent variables, and the stability of ELM is the response value. The fitted model of R^2^, adjusted R^2^, and predicted R^2^ are 0.9951, 0.9901, and 0.9733, respectively. The predicted R^2^ and adjusted R^2^ are both near to 1, indicating that the model equation fits well. The fitting equation can be used to determine the preparation conditions of a stable ELM by adjusting the parameters. Adeq Precision is the signal to noise ratio by comparing the predicted value range at the design point to the average prediction error. The model has adequate discriminative capacity if the ratio is greater than 4. The Adeq Precision of the model obtained in this experiment is 56.4267.

(10) $$S=8.05-1.16\times A-0.32\times B+0.46\times C-0.0010\times D-0.0046\times AB+0.00064\times AC\;\begin{array}{l} +0.000024\times AD-0.020\times BC+4.17\times 10^{-6}\times BD+4.38\times 10^{-6}\times CD\\ +0.069\times A^{2}+0.022\times B^{2}-0.00074\times C^{2}+6.29\times 10^{-8}\times D^{2} \end{array}$$

The significance of the model was evaluated using analysis of variance (ANOVA), and the specific analysis results are shown in Table 3. The F-value and *p*-value are used to assess the regression. The model in this study has an F-value of 201.69 and a *p*-value of less than 0.0001, indicating that the regression is highly significant. The *p*-values for the linear variables *A*, *B*, *C*, and *D* in the model are less than 0.0001, demonstrating that the four linear variables have significant impact on the model. The significant effects on the stability of the ELM were *AD* and *BC* (*p*-value < 0.0001), while the other interaction variables were insignificant effects (*p*-value > 0.05). Table 3 shows that the *p*-value of the model is less than 0.0001, indicating the residual lack of fit is insignificant and the model fulfills the fitting requirement.

### 3.2. Effects of Different Variables on the Stability of WELM

Because the removal rate of phenol is not the main goal of the research, it is not shown in further detail. The removal rates of phenol in purified water of sour water stripper by WELM were in the range of 85–93%, and the residual phenol concentration were about 25–33 mg/L.

**Figure 4:**
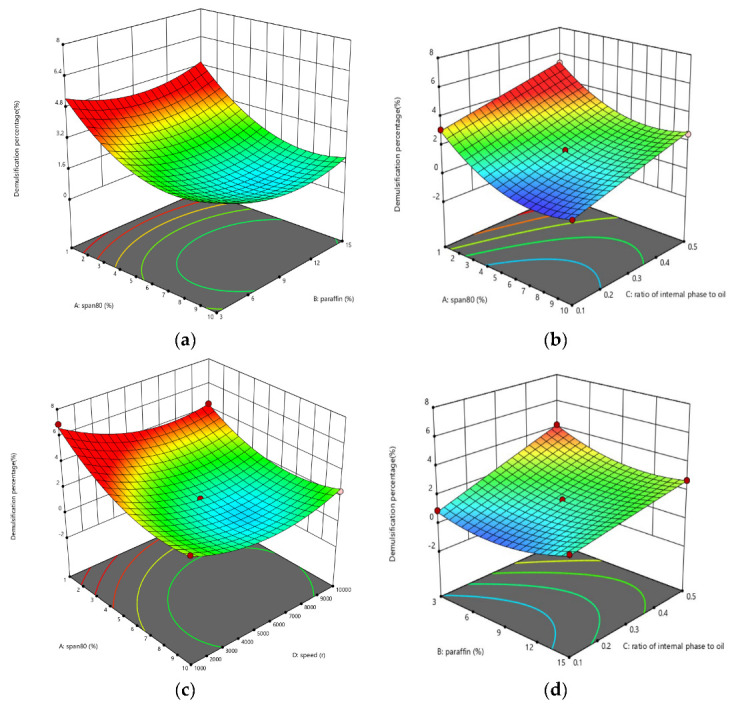

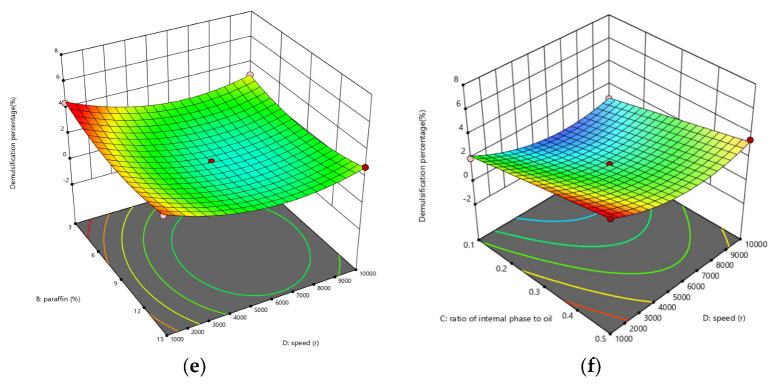
(**a**) Concentration of Span-80 and liquid paraffin; (**b**) concentration of Span–80, ratio of internal phase to oil; (**c**) concentration of Span-80, rotational speed of homogenizer; (**d**)concentration of liquid paraffin, ratio of internal phase to oil; (**e**) concentration of liquid paraffin, rotational speed of homogenizer; (**f**) ratio of internal phase to oi, rotational speed of homogenizer.

The interaction effects of variables on the stability of WELM (demulsification rate) are shown in Figure 4. The higher the eccentricity of response surface, the greater the significance of variable interaction. The interaction of A-Span-80 and B-liquid paraffin concentrations were not significant, as shown in Figure 4a. The Span-80 surfactant is crucial in the production of WELM as well as the extraction and separation process. Surfactant absorbed at the phase interface, reducing interfacial tension between oil and water, and improving WELM stability. Surfactant hydrophilic-lipophilic balance (HLB) represents the degree of interaction and force balance between the hydrophilic and lipophilic groups of the molecule. When HLB is in the range of 1~10, surfactant solubility in the oil phase is greater than in the water phase, making it simple to form a W/O type emulsion. The surfactant used in this study (Span-80) has an HLB value of 4.3, which facilitates the formation of a W/O emulsion film. Under a certain concentration of liquid paraffin, the demulsification rate of WELM decreases as the surfactant concentration increases, and the variation is stable after a certain concentration is reached. The tendency is the same for different liquid paraffin concentrations, demonstrating that there is no significant interaction between Span-80 concentration and liquid paraffin concentration. The higher the surfactant concentration, the denser the arrangement at the interface, resulting in a stronger adsorption layer [17,18].

The interactive effect of Span-80(A) concentration and internal-oil ratio(C) is shown in Figure 4b. The interactive effect of A and C is negligible. In different concentrations of Span-80, the demulsification rates increase with the increasing of internal-oil ratio and the WELM stability is low. The particle size of the internal droplets gets larger as the fraction of internal phase increases, the thickness and viscosity of the WELM decreases, and the stability of the ELM reduces [19,20,21].

The interactive effect of Span-80(A) concentration and rotational speed of homogenizer (D) on demulsification rate is shown in Figure 4c and the interactive effect is significant. The demulsification rates decrease with A increasing at the low level of D. The effect of A on the demulsification rate is relatively slight, but the changing trend is the same as when D concentration is low. The demulsification rates decrease with an increase in D first. When D increases to more than 6000 rpm, the demulsification rate increases. In conclusion, increasing the concentration of surfactant can considerably improve WELM stability [22,23,24,25]. The provided shear force and mechanical energy increase with the homogenizer rotational speed increasing, and a smaller particle size of WELM can be produced, leading to a more stable WELM. On the other hand, excessive shear force can increase the internal energy of the liquid film, raise the temperature, and induce the WELM particles to move violently, leading WELM to break. If the shear force is too great, the ELM will be destroyed as well [26]. The factor D has a significant effect on the demulsification rate when A is low. And the effect of D on the demulsification rate is negligible at a high level of A. With the increase in A, the demulsification rate gradually decreases [27,28].

The interactive effect of liquid paraffin concentration (B) and internal-oil ratio (C) on demulsification rate is shown in Figure 4d, and the interactive effect is significant. The demulsification rates increase as C increases. When C is low, the influence of B on demulsification rate is negligible. When C is at a high level, the demulsification rates reduce as B increases. The liquid film thickness is primarily affected by C. The greater the C, the thinner the WELM and the less stable it is. As a result, decreasing C leads to an increase in WELM stability [29]. The demulsification rate of WELM increases with the increase in C, and increasing the concentration of liquid paraffin in WELM can reduce its fluidity and improve its stability, leading to a reduction of demulsification rate. The WELM is stable when the internal-oil ratio is low, and the effect of liquid paraffin concentration on the demulsification rate is negligible [30,31].

The interactive effect of liquid paraffin concentration (B) and rotational speed of homogenizer (D) on demulsification rate is shown in Figure 4e, and the interactive effect is negligible. Demulsification rates decrease as D increases. When D raises to more than 6000 rpm, the increase in D will reduce the stability of WELM. The demulsification rate decrease slightly with the increasing of B. The influence of B is more evident when D is at a low level.

The interactive effect of internal-oil ratio (C) and rotational speed of homogenizer (D) on demulsification rate is shown in Figure 4f, and the interactive effect is slight. The demulsification rate decrease with the increase in D. When D increases to 4000–5000 rpm, with the increase in D, the demulsification rate increases. And the increasing of internal-oil ratio leads to the reduction of WELM stability.

In conclusion, only the interaction effects on demulsification rate between A and D, B and C are significant.

### 3.3. ANN Modeling and Assessment

According to the Equations (3) and (4), the number of neurons in the input layer and the output layer are 4 and 1, respectively. The suitable number of neurons in the hidden layer is in range of 4–10. The assessment results of the established ANN model are shown in Table 4.

**Figure 5:**
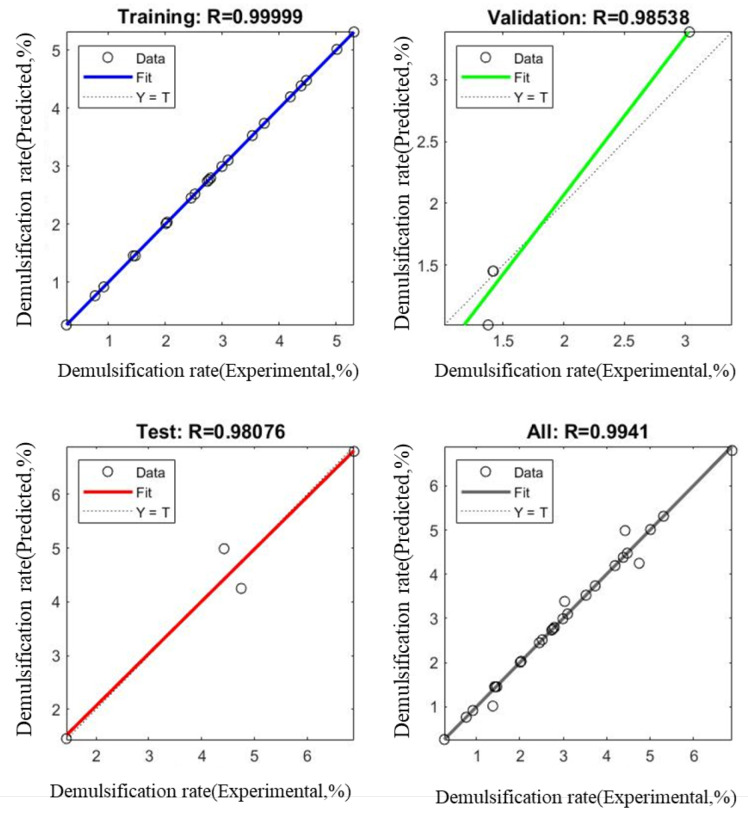
Regression diagram of ANN model with 10 hidden layer neurons.

When the number of neurons in the hidden layer is set to 10, the maximum R value of all data is 0.9941, and the MSE is 0.0000309. The experimental values of the demulsification rate are related to the model values, and the number of neurons in the hidden layer of the model is obtained by recurrent training. The R values of the derived model are more than 0.96. Figure 5 shows a linear regression relationship between the experimental values of the demulsification rate and the ANN model values, which includes the training set, validation set, test set, and total data. The total data R is 0.9941, indicating that the experimental values correlate well with the model values and the model has a good fitting and accuracy. Due to the limited amount of data, the R value in the validation set is relatively low. The model values obtained by the ANN model are shown in Table 5.

## 4. Comparison of RSM and ANN Models

**Figure 6:**
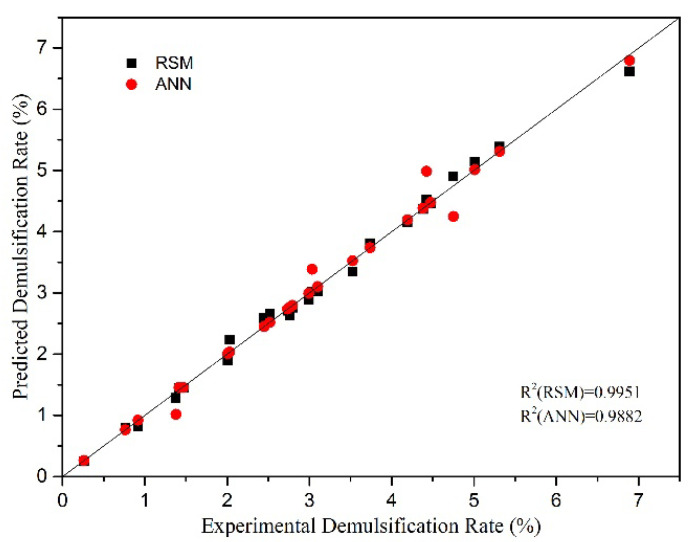
Comparison between the predicted values of RSM and ANN models and the experimental values of demulsification percentage.

The diagonal plots of model values and experimental values of the WELM demulsification rate determined are shown in Figure 6, and the model values are obtained by RSM and ANN models. The R values of the two models are also shown in Figure 6. The higher the R value, the better correlation between the experimental and model values. In comparison, R(RSM) > R(ANN), suggesting that the RSM model is more predictable than ANN model. The values of R^2^, RMSE and MAE regression are used to compare the predicted values obtained by the two models in Table 6. R^2^ is a metric that assess how well a model fits and is proportional to the degree of fitting. Outliers effect RMSE value more than MAE value, which is a quantitative measurement of the difference between model value and experimental value. In comparison, the R^2^ of RSM and ANN models are 0.9951 and 0.9882, respectively, indicating that both models have a good fitting degree. In addition, R2(RSM) > R2(ANN), RMSE(RSM) < RMSE(ANN), which means the RSM model is more predictable. And MAE(RSM) > MAE(ANN), suggesting that there may be outliers in the data.

The optimal condition parameters were obtained by RSM. The rotational speed of homogenizer was 4100 rpm, the of Span-80 was 9.4 wt.%, the liquid paraffin concentration was 10.2% (*v/v*), and the internal-oil ratio was 0.385. The RSM model and ANN model are used to predict the demulsification rate of the WELM under optimal condition and the experimental results are shown in Table 7. It can be seen that the demulsification rate of WELM is only 0.481%, and the stability is high. In addition, the prediction accuracy of the RSM model is relatively higher. And the WELM was employed to treat the purified water of sour water stripper, and the initial concentration of phenol is 178 mg/L, and the removal rate is 89.8% in the optimal condition.

Both RSM and ANN models have good predictability for the demulsification rate of WELM. The main differences between RSM and ANN models are as follows:

1. Before establishing RSM model, experiments must be designed and analyzed to obtain the model. The ANN model can be established based on the data that is already available.
2. The interaction effects and degrees of multiple variable factors can be obtained through the establishment of the RSM model. However, the establishment of the ANN model is a “black box” model establishment process, and it is difficult to compare and analyze the effect of variables on the results intuitively.
3. The RSM model can be obtained with fewer experimental data. The predictability of the model established by the ANN method will be significantly improved when the input experimental data increased.

## 5. Conclusions

In this study, the WELM was used to treat purified water of sour water stripper in an oil refinery facility, and waste oil from the oily sludge hydrothermal treatment. The WELM with highly efficient removal of phenol from wastewater consist of waste oil. The response surface method was employed to determine the effect of the single factor and interaction factors on the WELM stability. Meanwhile, the RSM and ANN model were established.

1. The concentration of Span-80, liquid paraffin, internal-oil ratio, and the rotational speed of homogenizer have significant effect on the demulsification rate of the WELM.
2. The removal rate of phenol from purified water of sour water stripper by WELM were higher than 85%.
3. The 3D response surface figures of the variables interactive effect on the demulsification rate were obtained by response surface analysis, and combined with the *p* value in the analysis of variance table, it can be found that the interaction effect between the concentration of Span-80 and the rotational speed of homogenizer, the concentration of liquid paraffin and the internal-oil ratio on demulsification rate are significant, and other interaction effects are negligible.
4. The RSM and ANN models have predictability for the demulsification rate of the ELM, and the RSM model is better. Under the optimal conditions, the demulsification rate is only 0.481%, which shows high stability and provides the best conditions for subsequent WELM recycling.

## Figures and Tables

**Table 1:** Properties of waste oil.

Properties	Water Content (%)	Flash Point (°C)	Ignition Temperature (°C)	Condensation Point (°C)	Mechanical Impurities (%)
Value	0.76	94	98	18	0.47

**Table 2:** Factor variables and different level values in BBD design.

Variables	Code	Levels		
		−1	0	1
Span-80 (wt.%)	Z_1_	1	5.5	10
liquid paraffin ((%(*v/v*))	Z_2_	3	9	15
internal-oil ratio	Z_3_	2	6	10
rotational speed of homogenizer (rpm)	Z_4_	1000	5500	10,000

**Table 3:** ANOVA analysis of RSM model with BBD design.

Source	Sum of Squares	f	Mean Square	F-Value	*p*-Value
Model	69.28	4	4.95	201.69	<0.0001
*A*-span80	22.56	1	22.56	919.45	<0.0001
*B*-liquid paraffin	0.7201	1	0.7201	29.35	<0.0001
*C*-internal-oil ratio	17.05	1	17.05	695.08	<0.0001
*D*-speed	4.81	1	4.81	196.18	<0.0001
*AB*	0.0613	1	0.0613	2.50	0.1361
*AC*	0.0005	1	0.0005	0.0220	0.8842
*AD*	0.9652	1	0.9652	39.34	<0.0001
*BC*	0.9196	1	0.9196	37.48	<0.0001
*BD*	0.0507	1	0.0507	2.06	0.1727
*CD*	0.0249	1	0.0249	1.01	0.3311
*A* ^2^	12.56	1	12.56	511.81	<0.0001
*B* ^2^	4.20	1	4.20	171.36	<0.0001
*C* ^2^	0.0009	1	0.0009	0.0373	0.8496
*D* ^2^	10.53	1	10.53	429.26	<0.0001
Residual	0.3435	1	0.0245	-	-
Lack of Fit	0.3148	0	0.0315	4.40	0.0832
Pure Error	0.0286	4	0.0072	-	-
Cor Total	69.62	8	-	-	-

**Table 4:** Comparison of results for different numbers of neurons in hidden layers.

Number of Hidden Layer Neurons	MSE (The Best Validation Performance)	R (All Data)
4	0.0252	0.97041
5	0.0000846	0.96366
6	0.0000580	0.98383
7	0.0000632	0.98697
8	0.0000632	0.97247
9	0.0000845	0.9765
10	0.0000309	0.9941

**Table 5:** Experimental value of the demulsification rate of the emulsion film and the predicted value of the RSM and ANN models.

Operation	A	B	C	D
	Span-80 (wt%)	Liquid Paraffin (%*v/v*)	Internal-Oil Ratio	Rotational Speed of Homogenizer (rpm)
1	5.5	9	10	1000
2	5.5	9	6	5500
3	5.5	9	6	5500
4	1	15	6	5500
5	5.5	3	6	1000
6	5.5	15	2	5500
7	5.5	15	6	10,000
8	5.5	9	6	5500
9	5.5	9	10	10,000
10	5.5	3	6	10,000
11	5.5	9	2	10,000
12	5.5	15	10	5500
13	1	9	10	5500
14	5.5	9	2	1000
15	1	9	6	10,000
16	1	9	6	1000
17	10	9	2	5500
18	10	15	6	5500
19	10	9	6	1000
20	5.5	9	6	5500
21	5.5	9	6	5500
22	10	3	6	5500
23	10	9	6	10,000
24	5.5	15	6	1000
25	1	3	6	5500
26	5.5	3	2	5500
27	5.5	3	10	5500
28	1	9	2	5500
29	10	9	10	5500

**Table 6:** Comparison of RSM and ANN demulsification percentage models.

Model	RSM	ANN
R^2^	0.9951	0.9882
RMSE	0.1050	0.1694
MAE	0.08178	0.06847

**Table 7:** Comparison of predicted and experimental values of emulsion film stability under optimal conditions.

Model	S(%)	Error(%)
RSM	0.536	11.4%
ANN	0.545	13.3%
Experimental value	0.481	-

## Data Availability

The data presented in this study are available in this article.

## References

[1] Qi Y.B., Luo C., Zhang S.J. (2021). Research progress on dephenolization technologies of wastewater containing phenolic compounds by liquid membrane. Chem. Ind. Eng. Prog..

[2] Ng Y.S., Jayakumar N.S., Hashim M.A. (2010). Performance evaluation of organic emulsion liquid membrane on phenol removal. J. Hazard. Mater..

[3] Yan B.-C., Zhang Y.-Y., Jiao X.-S., Chen K., Huang X.-J., Yin J.-C., Wang L. (2020). Advances in research on structural characteristics and stability of emulsion membranes. Appl. Chem. Ind..

[4] Ahmad A.L., Kusumastuti A., Derek C., Ooi B.S. (2012). Emulsion liquid membrane for cadmium removal: Studies on emulsion diameter and stability. Desalination.

[5] Sulaiman R., Othman N., Amin N., Othman N., Amin N. (2014). Emulsion liquid membrane stability in the extraction of ionized nanosilver from wash water. J. Ind. Eng. Chem..

[6] Abbassian K., Kargari A. (2014). Effect of polymer addition to membrane phase to improve the stability of emulsion liquid membrane for phenol pertraction. Desalin. Water Treat..

[7] Sujatha S., Rajamohan N., Vasseghian Y., Rajasimman M. (2021). Conversion of waste cooking oil into value-added emulsion liquid membrane for enhanced extraction of lead: Performance evaluation and optimization. Chemosphere.

[8] Othman N., Shu L.Y., Ooi Z.Y., Jusoh N., Idroas M., Goto M. (2017). Easy removing of phenol from wastewater using vegetable oil-based organic solvent in emulsion liquid membrane process. Chin. J. Chem. Eng..

[9] Tahmasebizadeh P., Javanshir S., Ahmadi A. (2021). Zinc extraction from a bioleaching solution by emulsion liquid membrane technique. Sep. Purif. Technol..

[10] Arun V., Jalaludeen S.S., Jayakumar S., Swaminathan S. (2021). Effect of contacting pattern and various surfactants on phenol extraction efficiency using emulsion liquid membrane. Int. J. Chem. React. Eng..

[11] Djenouhat M., Hamdaoui O., Chiha M., Samar M.H. (2008). Ultrasonication-assisted preparation of water-in-oil emulsions and application to the removal of cationic dyes from water by emulsion liquid membrane. Sep. Purif. Technol..

[12] Mortaheb H.R., Amini M.H., Sadeghian F., Mokhtarani B., Daneshyar H. (2008). Study on a new surfactant for removal of phenol from wastewater by emulsion liquid membrane. J. Hazard. Mater..

[13] Salman H.M., Mohammed A.A. (2019). Extraction of lead ions from aqueous solution by co-stabilization mechanisms of magnetic Fe_2_O_3_ particles and nonionic surfactants in emulsion liquid membrane. Colloids Surf. A Physicochem. Eng. Asp..

[14] Kumar A., Avinash T., Panesar P.S. (2019). Recent developments on sustainable solvents for emulsion liquid membrane processes. J. Clean. Prod..

[15] Shokri A., Daraei P., Zereshki S. (2020). Water decolorization using waste cooking oil: An optimized green emulsion liquid membrane by RSM. J. Water Process Eng..

[16] Mohammadi F., Samaei M.R., Azhdarpoor A., Teiri H., Badeenezhad A., Rostami S. (2019). Modelling and optimizing pyrene removal from the soil by phytoremediation using response surface methodology, artificial neural networks, and genetic algorithm. Chemosphere.

[17] Daas A., Hamdaoui O. (2010). Extraction of bisphenol A from aqueous solutions by emulsion liquid membrane. J. Hazard. Mater..

[18] Mohammed S.A.M., Zouli N., Al-Dahhan M.M., Zouli N., Al-Dahhan M. (2018). Removal of phenolic compounds from synthesized produced water by emulsion liquid membrane stabilized by the combination of surfactant and ionic liquid. Desalin. Water Treat..

[19] Lin Z., Zhang Z., Li Y., Deng Y. (2016). Magnetic nano-Fe_3_O_4_ stabilized Pickering emulsion liquid membrane for selective extraction and separation. Chem. Eng. J..

[20] Reis M.T.A., Freitas O.M., Agarwal S., Ferreira L.M., Ismael M.R.C., Machado R., Carvalho J.M. (2011). Removal of phenols from aqueous solutions by emulsion liquid membranes. J. Hazard. Mater..

[21] Hussein M.A., Mohammed A.A., Atiya M.A., Mohammed A.A., Atiya M.A. (2019). Application of emulsion and Pickering emulsion liquid membrane technique for wastewater treatment: An overview. Environ. Sci. Pollut. Res..

[22] Kumar A., Thakur A., Panesar P.S. (2019). A review on emulsion liquid membrane (ELM) for the treatment of various industrial effluent streams. Rev. Environ. Sci. Biotechnol..

[23] Bensalah N., Midassi S., Ahmad M.I., Bedoui A. (2020). Degradation of hydroxychloroquine by electrochemical advanced oxidation processes. Chem. Eng. J..

[24] Jusoh N., Othman N., Nasruddin N.A. (2016). Emulsion liquid membrane technology in organic acid purification. Malays. Soc. Anal. Sci..

[25] Kohli H.P., Gupta S., Chakraborty M. (2018). Extraction of Ethylparaben by emulsion liquid membrane: Statistical analysis of operating parameters. Colloids Surf. A Physicochem. Eng. Asp..

[26] Mohamed Noah N.F., Jusoh N., Othman N., Raja Sulaiman R.N., Parker N.A.M.K. (2018). Development of stable green emulsion liquid membrane process via liquid–liquid extraction to treat real chromium from rinse electroplating wastewater. J. Ind. Eng. Chem..

[27] Kumar A., Thakur A., Panesar P.S. (2019). A comparative study on experimental and response surface optimization of lactic acid synergistic extraction using green emulsion liquid membrane. Sep. Purif. Technol..

[28] Raval A.R., Kohli H.P., Mahadwad O.K. (2022). A Comprehensive Review on Green Emulsion Liquid Membrane and Its Applicability Towards the Removal of Contaminants from the Aquatic Streams. Water Air Soil Pollut..

[29] Ahmad A.L., Zaulkiflee N.D., Kusumastuti A., Shah Buddin M.M.H. (2019). Removal of Acetaminophen from Aqueous Solution by Emulsion Liquid Membrane: Emulsion Stability Study. Ind. Eng. Chem. Res..

[30] Al-Obaidi O., Alabdulmuhsin M., Tolstik A., Trautman J.G., Al-Dahhan M. (2021). Removal of hydrocarbons of 4-Nitrophenol by emulsion liquid membrane (ELM) using magnetic Fe_2_O_3_ nanoparticles and ionic liquid. J. Water Process Eng..

[31] Zeng L., Liu Y., Yang T., Yang Y., Tang K. (2018). Simultaneously enhanced ELM selectivity and stability by difunctional additives for batch and continuous separation of Cd(II)/Cu(II). Chem. Eng. Res. Des..

